# Groundwater Contaminated with Hexavalent Chromium [Cr (VI)]: A Health Survey and Clinical Examination of Community Inhabitants (Kanpur, India)

**DOI:** 10.1371/journal.pone.0047877

**Published:** 2012-10-24

**Authors:** Priti Sharma, Vipin Bihari, Sudhir K. Agarwal, Vipin Verma, Chandrasekharan N. Kesavachandran, Balram S. Pangtey, Neeraj Mathur, Kunwar Pal Singh, Mithlesh Srivastava, Sudhir K. Goel

**Affiliations:** 1 Petroleum Toxicology Division, CSIR-Indian Institute of Toxicology Research (CSIR-IITR), Lucknow, India; 2 Epidemiology Division, CSIR-IITR, Lucknow, India; 3 Department of Biochemistry, Lucknow University, Lucknow, India; 4 Environmental Chemistry, CSIR-IITR, Lucknow, India; 5 Pesticide Toxicology Division, CSIR-IITR, Lucknow, India; California Pacific Medicial Center Research Institute, United States of America

## Abstract

**Background:**

We assessed the health effects of hexavalent chromium groundwater contamination (from tanneries and chrome sulfate manufacturing) in Kanpur, India.

**Methods:**

The health status of residents living in areas with high Cr (VI) groundwater contamination (N = 186) were compared to residents with similar social and demographic features living in communities having no elevated Cr (VI) levels (N = 230). Subjects were recruited at health camps in both the areas. Health status was evaluated with health questionnaires, spirometry and blood hematology measures. Cr (VI) was measured in groundwater samples by diphenylcarbazide reagent method.

**Results:**

Residents from communities with known Cr (VI) contamination had more self-reports of digestive and dermatological disorders and hematological abnormalities. GI distress was reported in 39.2% vs. 17.2% males (AOR = 3.1) and 39.3% vs. 21% females (AOR = 2.44); skin abnormalities in 24.5% vs. 9.2% males (AOR = 3.48) and 25% vs. 4.9% females (AOR = 6.57). Residents from affected communities had greater RBCs (among 30.7% males and 46.1% females), lower MCVs (among 62.8% males) and less platelets (among 68% males and 72% females) than matched controls. There were no differences in leucocytes count and spirometry parameters.

**Conclusions:**

Living in communities with Cr (VI) groundwater is associated with gastrointestinal and dermatological complaints and abnormal hematological function. Limitations of this study include small sample size and the lack of long term follow-up.

## Introduction

Chromium (Cr) is listed by the Environmental Protection Agency as one of the 129 priority pollutants and one of the 14 most noxious heavy metals. Exposure to Cr (VI) via inhalation route has been declared as carcinogenic by various agencies [1], [2]. Levels above permissible limit have also been found at various residential areas [3], [4], [5], [6], [7]. Recently, Environmental Working Group, USA in 2010 reported that 89% water samples from cities in America had hexavalent chromium [Cr (VI)] levels much higher than California Safety Standards [8]. Despite this, Cr (VI) contaminated water has not yet attained a well-defined status of being toxic or safe. This is due to the limited number of studies on general population getting exposed to this metal.

The unique anti-corrosive and tanning properties of Cr favor its widespread application in chrome plating industries, leather tanneries, etc. However, not many industries follow norms of treating toxic waste before its release into the environment. This has resulted in increased ecological toxic burden leading to groundwater contamination. According to a report by Tata Environmental Research Institute, India, out of 7.2 million tons of hazardous waste from industries generated each year in India, 5.2 million tons is improperly disposed off [9]. Kanpur, one of the most industrialized cities of India, is a hub of tanneries and the industries manufacturing basic chrome sulphate (BCS). While the records of Central Leather Research Institute, India showed only 170 tanneries at Kanpur, a study conducted in 2000 found twice this number in just one tanning cluster [10]. Annually, these tanneries alone discharge more than 1500 metric tons of chromium sulphate as waste [11]. The waste from these industries has been illegally dumped in deep borings, open lands and in rivers through decades [7], [12]. In 1997, Central Pollution Control Board, India reported Cr (VI) concentration upto 250 times higher than the WHO permissible limit (0.05 ppm) in areas at Kanpur [13]. A recent report was also in agreement with this [14]. Understanding the extent of this problem, Blacksmith institute, a non-profitable international organization has included Kanpur among list of the most polluted places in world [15]. Apparently, bids for the removal of hazardous wastes dumped illegally have been made; however, this problem is still unresolved [16].

We realized that ever since this unseemly sight of yellow water was noticed [3], [4], questions concerning its impact on humans have been raised. It is of relevance to find out health risk to the residents in these contaminated areas. Thus, we undertook population health assessment to know health risk to the residents from areas of Kanpur having Cr (VI) contaminated groundwater. Health status was assessed through self-reported health questionnaires, general medical examination, spirometric analysis and blood hematology measures.

## Materials and Methods

### Study Design

**Figure 1:**
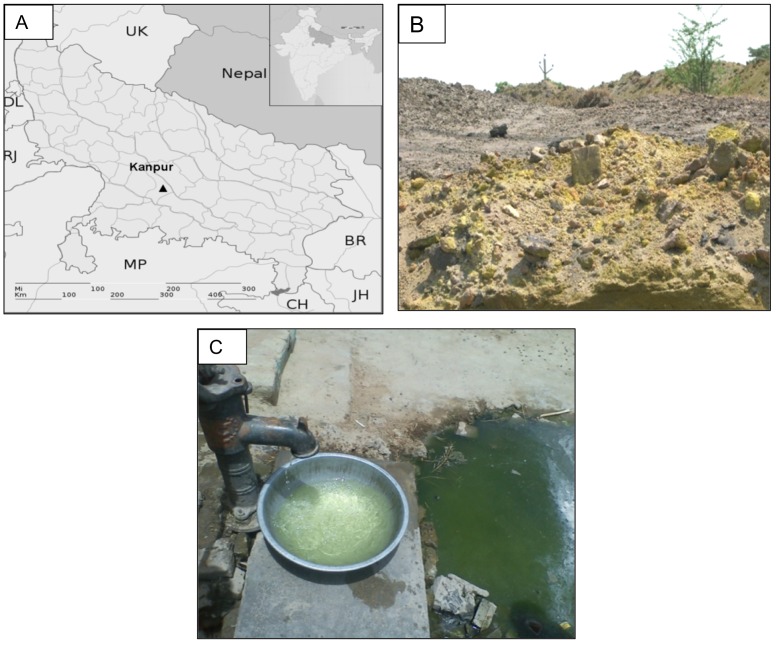
Selected characteristics of the study location. (A) Map showing city of Kanpur- Figure in inset is map of India showing Uttar Pradesh state (U.P.) (dark filled). Surrounding states: UK-Uttarakhand, HR- Haryana, DL- Delhi, RJ- Rajasthan, MP- Madhya Pradesh, CH- Chattisgarh, JR- Jharkhand, BR- Bihar and far-east country Nepal; (B) Photograph of a chrome sludge dumping site at Kanpur (C) Photograph showing yellow colored contaminated water from a handpump.

A cross-sectional retrospective study was carried out on the general population residing at Kanpur, a city in Uttar Pradesh, India (26.4670° North and 80.3500°East) (Figure 1A). We organized health camps among contaminated and non-contaminated communities to collect data on general health status of the residents. The permission and local assistance for arranging camps were obtained through meetings with authorized persons like village headman (gram- pradhan) and corporator. The Institutional Human Ethics Committee of IITR granted ethical clearance for this study.

### Selection of the Sites

The contaminated sites (as shown in Figure 1B) were selected based on the earlier reports by CPCB [7], [13] showing high level of Cr (VI) in groundwater (WHO permissible limit: >0.05 ppm). To further confirm the present status, groundwater samples were collected from handpumps (as shown in Figure 1C) and deep borings and Cr (VI) was estimated in them using standard diphenylcarbazide reagent method [17]. A control population having similar socio-demographic status was also included as a selected reference community. This reference community is from Kanpur where neither in any previous report nor by current estimation, Cr (VI) level in groundwater was found to be elevated.

### Selection Criteria of the Subjects

Inclusion criteria- Individuals with age ≥18 years, with duration of residence ≥1 year and not consuming bottled water were included irrespective of their health status.

Exclusion criteria- Individuals with age <18 years, migratory population, with duration of residence <1 year and having occupational exposure to chromium compounds which includes the occupations where Cr compounds are used, namely, workers at leather tanneries, chrome sulphate manufacturing units, paint/dye synthesis and chrome plating industries.

### Recording of Information and General Medical Examination

We organized health camps on dates approved by the gram pradhan. A written informed consent was obtained from each subject; however, the subjects were free to withdraw their participation at any point of time. A pre-tested questionnaire was completed by interviewing the subjects. Simultaneously, a medical scientist examined the subjects and evaluated complaints in order to avoid the chance of misreporting. The medical examination was done in accordance with the recommendations outlined in the Declaration of Helsinki [18]. Self reported heath information related to the occurrence of systemic health complaints (a strong predictor of long-term morbidity) [19] was gathered from the residents (Table S1). Information about the incidence of seasonal allergies and diagnosis of diabetes or asthma was also gathered. A female volunteer communicated with and assisted in collecting information from female participants due to social restrictions.

A physiologist conducted the lung-function test (LFT) using an electronic spirometer as per American Thoracic Society recommendations. LFT included forced expiratory volume after 1 second (FEV1) and peak expiratory flow rate (PEFR). Standard reference values for spirometry were taken from Rastogi’s prediction [20] which provided predicted ventilator norms for healthy and asymptomatic population.

### Blood Sample Collection

Blood samples were collected from interested subjects only. About 2 ml blood was withdrawn by venipuncture in EDTA coated vacutainers (BD Biosciences), mixed gently, stored in ice-cool boxes and transported to laboratory within 3–4 hours. Hematological parameters i.e., total leucocyte count (TLC), differential leucocyte count (DLC), red blood cell (RBC) count, mean corpuscular volume (MCV) and platelet count (PLT) were analyzed by MS-9 automated blood counter (France).

### Statistical Analyses

Descriptive statistics were generated to determine the distribution of socio-demographic characteristics among exposed and control group. Frequencies and percentages were shown for categorical variables. The main predictor variable considered was residence in Cr (VI) contaminated area. The other covariates were: age (years), dietary status (veg/non-veg), education level (illiterate/primary/high school & above), smoking habit (yes/no) and duration of residence (years). Occurrence of health complaint (yes/no) pertaining to different body systems was considered as main outcome variable. Separate models were constructed for separate outcome variables. Crude odds ratios (ORs) and 95% confidence intervals (CI) were calculated to analyze qualitative data (difference between proportions with complaints in exposed and control group) and statistical significance was tested by chi-square test. ORs were adjusted for potential covariates using multivariate logistic regression analysis. Models used to predict central nervous complaints (dizziness/feeling of faintness) and musculoskeletal complaints (numbness, tingling or weakness in limbs) also included self-reported diabetes as a covariate. Models predicting respiratory complaints (congestions, sore throat) also included self-reported allergies and asthma as covariates.

Mean values for quantitative parameters (hematology measures, spirometric parameters) among exposed and control group were compared using Student’s *t-test*. Prior to this, Levene’s test for equivalence of variance among the two groups was used to ascertain the homogeneity of variance. A 0.05 cutoff point was set for the *p*-value and applied in all statistical analyses. All analyses were carried out using SPSS 13.0.

## Results

### Self-Reported Health Complaints in Study Population

In all, 433 individuals participated in the health camps. Of these, 416 individuals were included in the study based on selection criteria. The study population was of Asian Indian origin. In particular, 186 exposed subjects consisting of 102 males and 84 females from the Cr (VI) contaminated communities participated. We found Cr (VI) levels upto 390 fold (20 ppm approx.) higher than the permissible limit in these areas. Control population from the reference community consisted of 230 subjects including 87 males and 143 females. Among exposed females, 14.3% were widows, 75% were married and rest were bachelor (10.7%). The respective frequencies for widows, married/single and bachelor among controls were 6.3%, 81.1% and 12.6%. Among males, 73.5% exposed and 78.2% control subjects were married and the rest were bachelor. It was observed that 97% exposed and 91% control population was using groundwater resources for various purposes. Duration of residence of the subjects from contaminated and reference communities ranged from 2–80 years and 1–60 years, respectively. Table 1 demonstrates the socio-demographic characteristics of study population stratified by gender.

**Table 1:** Socio-demographic characteristics of the study population stratified by gender.

		Control Population (N = 230)		Exposed Population (N = 186)	
		Malesn (%)	Femalesn (%)	Malesn (%)	Femalesn (%)
**Subjects**		87 (37.8)	143 (62.2)	102 (54.8)	84 (45.2)
**Age (years)**	Mean±SD	40.05±17.6	37.33±13.6	41.33±16.0	37.74±11.8
**Smoking habit**	Smokers^a^	17 (19.8)	0	37 (36.3)	1 (1.2)
**Education status**	Illiterate	7 (8)	22 (15.4)	23 (22.5)	38 (45.2)
	Primary	11 (12.6)	30 (21)	21 (20.6)	17 (20.2)
	High school	32 (36.8)	45 (31.5)	29 (28.4)	16 (19)
	Graduation & above	37 (42.5)	46 (32.2)	29 (28.4)	13 (15.4)
**Diet (Veg/Non-veg)**	Vegetarian	58 (66.7)	124 (86.7)	68 (66.7)	58 (69)

SD- Standard Deviation.

a Information regarding smoking status is missing for one male.

Prevalence for systemic health complaints such as gastrointestinal (GI) (*p* = 0.001), dermal (*p* = 0.001) (Figure S1), ocular (*p*<0.01) and urinary (*p*<0.01) among exposed population compared with controls is shown in Table 2. Table 3 presents gender-stratified crude odds ratios (COR) and adjusted odds ratios (AOR) for association between health complaints prevalence and residence at Cr (VI) contaminated communities. After adjustment for covariates, prevalence of GI, dermal and ocular complaints remained significant among exposed males. In females, urinary complaints along with GI and dermal were significantly higher. AOR for any symptom related to GI among exposed males and females were 3.1 (95% CI: 1.50–6.39) and 2.44 (95% CI: 1.32–4.52), respectively. For any skin related symptom, AOR among exposed males and females were 3.5 (95% CI: 1.41–8.58) and 6.57 (95% CI: 2.64–16.32), respectively with nearly 2 fold higher odds ratio in females compared to males (6.57 vs. 3.5). AOR for ocular complaints among exposed males was 3.5 (95% CI: 1.22–9.79) and for urinary complaints among females was 3.1 (95% CI: 1.08–8.87). Higher musculoskeletal complaints among controls became non- significant after adjustment.

**Table 2:** Prevalence of systemic health complaints among exposed population compared with controls.

**Systemic complaints**	**Control Population**			**Exposed Population**		
	**Males (N = 87)**	**Females (N = 143)**	**Total (N = 230)**	**Males (N = 102)**	**Females (N = 84)**	**Total (N = 186)**
Eyes	5 (5.7)	13 (9.1)	18 (7.8)	20 (19.6)**	20 (19.6)**	34 (18.2)**
Teeth and gums	28 (32.2)	47 (32.9)	76 (33)	42 (41.2)	42 (41.2)	78 (41.9)
Gastrointestinal	15 (17.2)	30 (21)	44 (19.1)	40 (39.2)***	40 (39.2)***	73 (39.3)***
Respiratory	16 (18.4)	22 (15.4)	38 (16.5)	23 (22.5)	23 (22.5)	33 (17.7)
Cardiovascular	15 (17.2)	24 (16.8)	39 (16.9)	13 (12.7)	13 (12.7)	24 (12.9)
Musculoskeletal	21 (24.1)	62 (43.4)	83 (36.1)	21 (20.6)	21 (20.6)	46 (24.7)*
Central Nervous	8 (9.2)	29 (20.3)	37 (16.1)	16 (15.7)	16 (15.7)	35 (18.8)
Urinary	5 (5.7)	6 (4.2)	11 (4.8)	13 (12.7)	13 (12.7)	23 (12.4)**
Dermal	8 (9.2)	7 (4.9)	15 (6.5)	25 (24.5)**	25 (24.5)**	46 (24.7)***
Eyes	5 (5.7)	13 (9.1)	18 (7.8)	20 (19.6)**	20 (19.6)**	34 (18.2)**

Numbers in parenthesis denote percentage.

* *p<0.05*,

** *p<0.01*,

*** *p<0.001*; significance tested by chi-square test.

**Table 3:** Crude odds ratios (COR) and adjusted odds ratios (AOR) for association between self-reported systemic health complaints and residence at Cr (VI) contaminated communities.

Systemic complaints	Males		Females	
	COR (95% CI)^a^	AOR (95% CI)	COR (95% CI)^a^	AOR (95% CI)
Eyes	4.00 (1.43–11.16)**	3.46 (1.22–9.79)^b^ *	2.00 (0.89–4.49)	2.20 (0.93–5.19)^b^
Teeth and gums	1.47 (0.81–2.68)	1.24 (0.66–2.33)^b^	1.53 (0.88–2.67)	1.54 (0.87–2.74)^b^
Gastrointestinal	3.10 (1.56–6.13)***	3.1 (1.50–6.39)^c^ **	2.44 (1.34–4.42)**	2.44 (1.32–4.52)^c^ *
Respiratory	1.29 (0.63–2.64)	1.21 (0.58–2.54)^d^	0.74 (0.33–1.66)	0.64 (0.27–1.54)^d^
Cardiovascular	0.70 (0.31–1.57)	0.59 (0.22–1.56)^b^	0.75 (0.35–1.61)	0.76 (0.32–1.80)^b^
Musculoskeletal	0.81 (0.41–1.62)	1.00 (0.47–2.14)^e^	0.55 (0.31–0.98)*	0.52 (0.26–1.02)^e^
Central Nervous	1.84 (0.74–4.53)	1.98 (0.71–5.52)^e^	1.15 (0.60–2.21)	1.20 (0.58–2.48)^e^
Urinary	2.39 (0.82–7.01)	2.14 (0.72–6.36)^b^	3.09 (1.08–8.82)*	3.09 (1.08–8.87)^b^ *
Dermal	3.21 (1.36–7.54)**	3.48 (1.41–8.58)^b^ **	6.48 (2.62–16.03)***	6.57 (2.64–16.32)^b^ ***

CI-confidence interval.

a Chi-square test was used to test significance for odds ratio. Control group from reference community is taken as reference category.

b Adjusted for age (continuous in years), smoking (yes/no) and education status (Illiterate/primary/high school/graduation & above).

c Adjusted for age (continuous in years), smoking (yes/no), education status (Illiterate/primary/high school/graduation & above) and diet (veg/non-veg).

d Adjusted for age (continuous in years), smoking (yes/no), education status (Illiterate/primary/high school/graduation & above), self-reported allergy and asthma.

e Adjusted for age (continuous in years), smoking (yes/no), education status (Illiterate/primary/high school/graduation & above) and self-reported diabetes.

* *p<0.05*,

** *p<0.01*,

*** *p<0.001.*

### Hematological Parameters

Analysis of blood samples from 70 control and 143 exposed subjects showed that there was no significant difference in TLC and DLC among exposed and control groups (data not shown). However, other hematology measures (RBC, PLT, MCV) showed significant difference among the two groups (Table 4). Among exposed subjects, RBC count was elevated (*p* = 0.001) in 30.7% males and 46.1% females while PLT was found to be markedly decreased (*p* = 0.001) in 68% males and 72% females. MCV was also lower (*p* = 0.001) among 62.8% males.

**Table 4:** Hematological alterations among exposed male and female subjects compared with the controls.

Parameters (unit)	Males			Females		
	Control (N = 30)	Exposed (N = 78)	t-value	Control (N = 40)	Exposed (N = 65)	t-value
**RBC** (10^6^/µl)	4.28±0.69	5.55**±**1.39***	4.79	3.89±0.71	5.67**±**1.26***	8.19
**MCV** (µm^3^)	85.38±7.89	78.56**±**9.18***	−3.37	83.24±9.27	82.81**±**9.1	−0.38
**PLT** (10^3^/µl)	190.3±59.3	116.2**±**42.9***	−7.38	228.4±76.9	120.2**±**56.5***	−8.3

RBC- Red Blood Cells count, MCV- Mean Corpuscular Volume, PLT- Platelets count.

Reference Range: RBC count- 4.3–6.2 (males) & 3.8–5.5 (females); MCV- 82–102 (males) & 78–101 (females); PLT- 150–450.

Values are shown as mean±SD where SD denote standard deviation.

* *p<0.05*,

** *p<0.01*,

*** *p<0.001*; *p* values adjusted for age and smoking by linear regression model.

### Lung Function Test

Lung function tests in 100 exposed and 70 control subjects showed no significant difference in FEV_1_ (1.48±0.53 vs. 1.58±0.65) and PEFR (247.78±116.1 vs. 281.31±97.42). There was no difference between exposed and unexposed in bronchial obstruction (11% vs. 4.3%), lung restriction (6% vs. 4.3%) and mixed ventricular defects (3% vs. 0%). However, overall spirometric abnormalities were higher among the exposed group (20% vs. 8.6%) (*p*<0.05).

## Discussion

This cross-sectional study reports higher prevalence of health complaints, altered hematological parameters and spirometric defects among inhabitants from Cr (VI) contaminated areas of Kanpur. Prevalence for GI complaints majorly included pain in abdomen, reduced appetite and diarrhea which are also reported by Zhang and Li [3] and Tokyo Metropolitan Government Bureau of Sanitation (TMBGS) [21]. The mortality based observational studies have also reported high incidence of death due to stomach cancer [3], [22], [23]. Further, chronic (2 years) animal study by NTP on rats and mice given Cr (VI) through drinking water reported incidences of neoplasm of the oral cavity and small intestine when compared to controls [24]. These reports strengthen our observation on GI distress among Cr (VI) exposed general population.

Further, the observation on skin abnormalities is supported by a survey at Tokyo (TMBGS) among residents from the Cr (VI) contaminated areas [21]. Some studies have also reported exacerbation of dermatitis in sensitized individuals by oral exposure to Cr (VI) [25], [26]. In the present study, Cr (VI) levels found in the contaminated area were also beyond the effective threshold limit of 10 ppm reported by Stern and his coworkers for elicitation of dermatitis [27]. We also found nearly 2 fold higher prevalence for dermal complaints among females when compared to males. The possible reason of more severity in females may be due to frequent exposure to Cr (VI) contaminated sources i.e., washing clothes, doing household cleaning and others jobs. Further, ocular complaints are supported by published reports on workers occupationally exposed to Cr (VI) [28], [29].

The hematological alterations, i.e., elevated RBC count, lowered MCV and PLT counts, have been reported in occupants residing close to tanneries [30]. The animal studies of Cr (VI) exposure through drinking water for 3 months [31] and 2 years [24] reported similar hematological alterations in rats. We hypothesize that the knowledge of water contamination made the residents to stick to insufficient drinking of water leading to observed higher RBC count and lower MCV among subjects from the contaminated areas. These clinical abnormalities may further have been aggravated due to diarrhea, as reported by the subjects.

High prevalence of respiratory illness [32] and mortality due to lung cancer [23] has been reported among population living in Cr (VI) contaminated areas. Reported spirometric abnormalities in the present study population may be one of the early events of respiratory disease development. We suggest that the defect in pulmonary function among studied population may become more pronounced in case, the exposure gets extended for longer duration.

Hexavalent chromium is suggested to be a major pollutant in the affected areas. A previous study found normal level of heavy metals (As, Cd, Cu, Fe, Mn, Ni, Pb, V and Zn), except Cr, in the groundwater from contaminated areas of Kanpur [33]. We also estimated in the groundwater samples Zn, Fe, Cd and As levels which were within acceptable limit, except Fe which was higher at contaminated and the reference community (unpublished observations). High intake of iron is reported to cause no adverse effects in humans [34]. Based on these observations, we suggest association between health adversities among inhabitants and the presence of Cr (VI) in groundwater.

There are also reports from Glasglow (UK) and New Jersey (USA) which are contrary to our observations [35], [36], [37], [38]. The possible factors for such discrepancies may be cultural and economic imbalance between developed and developing nations (improper waste management, malnutrition and lack of medical care facilities) [39]. These social-determinants may work in association with genetic predisposition factors. In this regard, we reported the significant role of *GSTM1* genetic polymorphism towards the dermal adversities in Cr (VI) exposed population [40]. The reasons toward differences in the sensitivity to Cr in males and females, involving genetic polymorphism are however, not evident. Thus, studies exploring the role of such variability factors are also needed along with the health assessment studies.

We cannot point out drinking of highly contaminated water as causative route of exposure to the participants. Other sources such as intake of contaminated diet and milk, use of contaminated water for taking bath and other cleaning work should also be considered while determining health risk to the subjects. This is supported by the reports on high levels of chromium in the agricultural produces from contaminated fields at Kanpur [41].

There are also certain limitations in the present study. These involve relatively small sample size and lack of long term follow-up among the residents. We further explain inadequacy of data in assessing relationship between occurrences of adversities and exposure period. Because no one knows exactly how long the ground water has been contaminated the duration of exposure may not be directly related to the period of residence and subsequent health outcomes. Although the first government report on the presence of Cr (VI) contamination of 16 ppm was in 1996 7, we are of view that this was not been an instant process. It seems more likely that a slow leaching process contaminated the aquifers, producing only small increments in consequences, thus delaying awareness and response by government agencies. This however, does not limit the significance of the prevalent health complications and associated symptoms found in the study population.

In conclusion, this retrospective study highlights the possibility of risk on human health through hexavalent chromium contaminated groundwater. The residents in contaminated areas were having higher prevalence of self-reports for gastrointestinal and skin ailments along with clinical alterations and spirometric defects. To prevent further damage to the public health and environment, actions on regulation of industrial waste management are needed in parallel with groundwater remedial measures.

## Supporting Information

Figure S1 **Photographs showing dermatological problems among subjects exposed to hexavalent chromium contaminated groundwater.**
(TIF)

Table S1 **Various self-reported health complaints included in the questionnaire.**
(DOC)
