# Supplementary figures and images for: Groundwater Contaminated with Hexavalent Chromium [Cr (VI)]: A Health Survey and Clinical Examination of Community Inhabitants (Kanpur, India)
Source: PLoS One. 2012 Oct 24;7(10):e47877. doi: 10.1371/journal.pone.0047877 (PMC3480439; doi:10.1371/journal.pone.0047877)

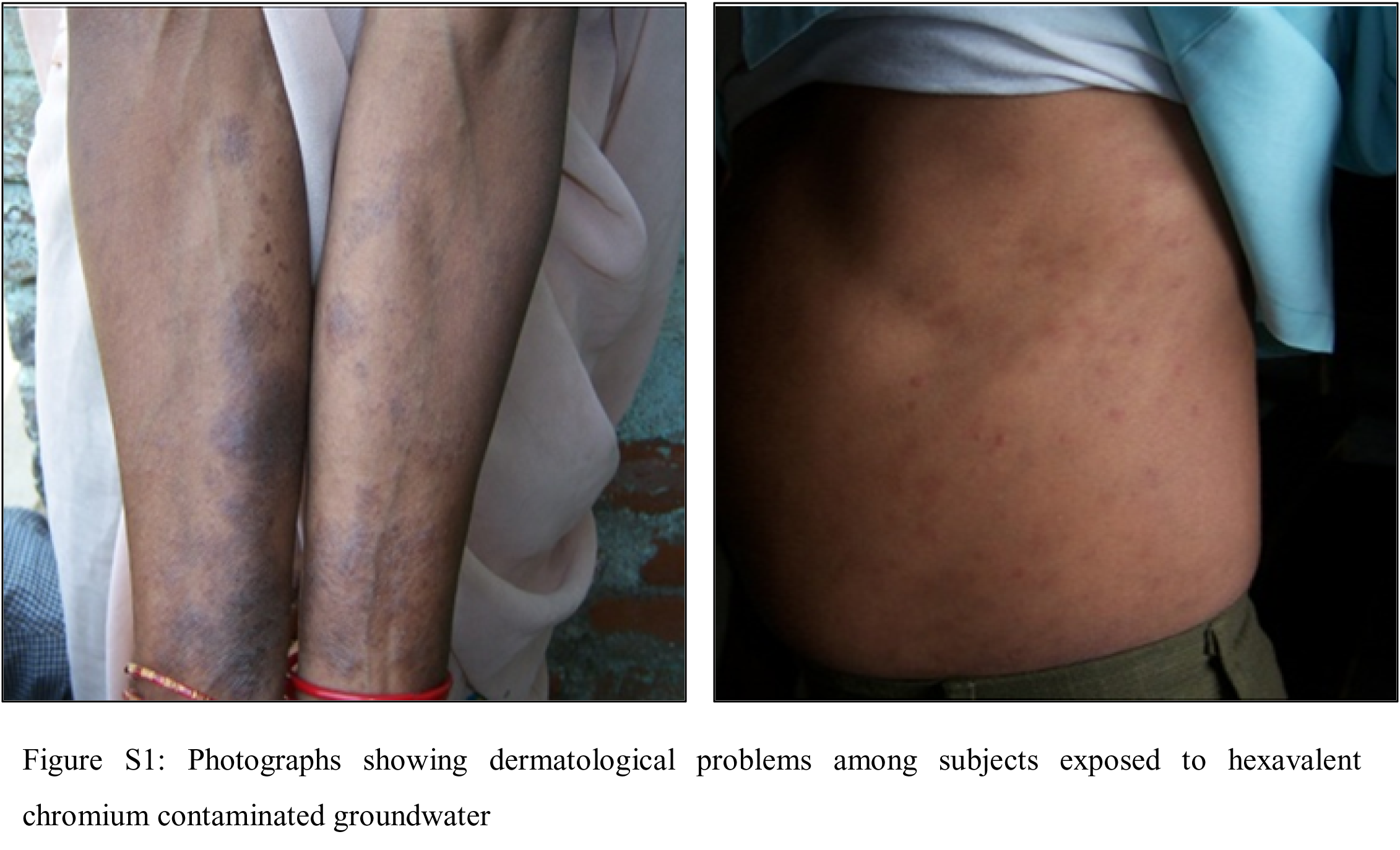

Supplement: Figure S1 — Photographs showing dermatological problems among subjects exposed to hexavalent chromium contaminated groundwater. (TIF) [file pone.0047877.s001.tif]
