# Supplementary material for: Groundwater Contaminated with Hexavalent Chromium [Cr (VI)]: A Health Survey and Clinical Examination of Community Inhabitants (Kanpur, India)
Source: PLoS One. 2012 Oct 24;7(10):e47877. doi: 10.1371/journal.pone.0047877 (PMC3480439; doi:10.1371/journal.pone.0047877)
Supplement: Table S1 — Various self-reported health complaints included in the questionnaire. (DOC) [file pone.0047877.s002.doc]

Table S1: Various self-reported health complaints included in the questionnaire.

| **System** | **Symptoms** | **Response** |
| --- | --- | --- |
| **Eyes** | Irritation, Itching, Cataract, Reduced vision | Yes/No |
| **Cardiovascular** | Hypertension, Hypotension, Pain in chest, Palpitation | Yes/No |
| **Teeth/gums/oral** | Toothache, Missing teeth, Discoloration,  Carious, Swollen/bleeding gums, Ulcers | Yes/No |
| **Gastrointestinal tract (GIT)** | Poor appetite, Stomach upset/indigestion, Gaseous discomfort, Diarrhoea/blood stained diarrhoea, Vomiting, Constipation, Stomach ulcers diagnosed by doctor | Yes/No |
| **Nervous** | Frequent severe headache, Frequently feel faint, Numbness or tingling in any part of body | Yes/No |
| **Urinary** | Urine output: reduced or increased, Burning while passing urine, Blood stained urine, Suffering from any kidney disorder | Yes/No |
| **Dermal** | Itching, Burning, Reddening, Crusting on skin of any area | Yes/No |
| **Respiratory** | Breathlessness, Cough, Tightness/Discomfort of chest | Yes/No |
| **Miscellaneous** | Diagnosed with asthma/allergy/diabetes/heart disease | Yes/No |
|  | Any other symptom? If yes, what? | Yes/No |
